# Image-Based Evaluation Method for the Shape Quality of Stacked Aggregates

**DOI:** 10.3390/s25237261

**Published:** 2025-11-28

**Authors:** Shaobo Ren, Sheng Zeng, Yi Zhou, Yuming Peng, Binqing Liu

**Affiliations:** 1 School of Civil Engineering, Chongqing Jiaotong University, Chongqing 400074, China; 611230080023@mails.cqjtu.edu.cn (S.R.); aleahcaswell143@gmail.com (Y.P.); 2 Guangxi Key Lab of Road Structure and Materials, Guangxi Transportation Science and Technology Group Co., Ltd., Nanning 530007, China; zengkun01@gmail.com; 3 School of Materials Science and Engineering, Chongqing Jiaotong University, Chongqing 400074, China; 622230980034@mails.cqjtu.edu.cn

**Keywords:** aggregate shape, digital image processing, stacked aggregates, shape index, standard deviation, evaluation standard

## Abstract

Coarse aggregate shape plays a critical role in determining surface performance and durability in pavement systems. Traditional manual shape inspection is laborious and subjective, especially for bulk aggregates in overlapped state. In this work, we propose an automated digital image-based evaluation method for stacked coarse aggregates, combining preprocessing (grayscale conversion, histogram equalization, Gaussian filtering), segmentation, and contour reconstruction via the Graham scan convex hull algorithm. Morphological parameters such as equivalent ellipse major/minor axes, area, and perimeter are then extracted to compute individual particle shape factors. To assess batch-level quality, shape factor standard deviations (σ) and mean shape factors were computed from 50 aggregate images. Comparison with manual measurement results shows mean relative errors below 15%. Our analysis reveals a strong correlation between σ and overall shape quality: lower σ indicates more uniform geometry, while higher σ suggests greater irregularity. Based on experimental data, we define three σ-based categories: excellent (σ ≤ 0.32), good (0.32 < σ ≤ 0.42), and poor (σ > 0.42). This σ-driven evaluation framework enables rapid, quantitative, and objective assessment of aggregate morphology in practical aggregate production and pavement quality control.

## 1. Introduction

Aggregate morphology, particularly particle shape, exerts a fundamental influence on the packing density, interlock, and mechanical performance of asphalt mixtures. Cubic aggregates improve stability and resistance to deformation, while flaky and elongated particles tend to fracture under compaction and loading, reducing mixture strength and durability, and ultimately accelerating pavement distress [1]. Accordingly, accurate characterization of aggregate shape is essential for quality control in pavement engineering. Beyond asphalt pavement applications, previous studies have shown that the morphology of mineral grains can also significantly influence the efficiency of separation processes in mineral beneficiation equipment, as well as the thermal and mechanical properties of preplaced aggregate concrete. These results further highlight the broad technological importance of accurate grain shape assessment in modern materials processing [2,3].

Conventional methods, such as flakiness and elongation indices, have been widely adopted in technical specifications and standards [4]. However, these approaches suffer from well-documented shortcomings: they are labor-intensive, time-consuming, and highly dependent on operator judgment, which reduces repeatability and objectivity [5]. More critically, these tests assess individual particles rather than capturing the variability of aggregate batches. Since aggregates are used and supplied in bulk, batch-level evaluation is vital for ensuring consistent quality in asphalt mixture production [6].

With the advancement of digital image processing, automated techniques have been increasingly applied to aggregate shape analysis [7]. Two-dimensional and three-dimensional imaging methods have been used to extract shape descriptors such as aspect ratio, circularity, angularity, and sphericity [8]. Studies have demonstrated that digital imaging provides reliable classification of cubic versus elongated particles and can quantify angularity with greater efficiency than manual approaches [9]. In addition, machine vision systems combined with statistical shape descriptors have been proposed to improve aggregate classification in laboratory and field applications [10]. Despite these advances, most existing studies have focused on single, manually isolated particles or carefully sieved specimens, while neglecting the stacked and bulk states that more closely reflect real production conditions. This gap has limited the practical application of image-based approaches in continuous quality control. However, when aggregates are present in a stacked state on conveyor belts, overlapping and occluded particles generate complex gray-level transitions and touching boundaries [11]. Most existing image-based systems implicitly assume well-separated or sparsely distributed particles; when directly applied to stacked aggregates they often merge adjacent grains into a single object, miss partially hidden edges, or produce unstable shape descriptors. These issues make it difficult to perform reliable, continuous monitoring of aggregate shape under realistic production conditions, and they constitute a key technical bottleneck for stacked aggregate detection.

Unlike previous studies, this research directly targets aggregates in a stacked state on conveyor belts, which represent the actual operational condition in production and construction environments. By addressing this scenario, the study bridges the gap between laboratory-based particle evaluation and field-scale aggregate quality monitoring [12].

To this end, a digital image-based framework is proposed with two major contributions. First, a robust image processing workflow—including grayscale conversion, histogram equalization, Gaussian filtering, watershed segmentation, and convex hull reconstruction—was established to extract accurate particle contours from stacked samples. Second, the standard deviation of the shape index (σk) is introduced as a batch-level metric to quantify shape uniformity. Together, these advances enable a two-tier evaluation framework, combining particle-level classification with σk-based batch assessment. The evaluation standard defines thresholds for σk corresponding to “Excellent,” “Good,” and “Poor” quality categories, and validation across multiple samples confirmed its robustness and reliability.

In summary, the novelty of this study lies in (i) extending digital image-based shape evaluation to stacked aggregates on conveyor belts, (ii) introducing σk as a batch-level uniformity parameter, and (iii) establishing a standardized evaluation framework applicable to real production environments. This work provides an objective, efficient, and scalable approach for aggregate quality control and contributes to the advancement of shape evaluation standardization in pavement engineering.

## 2. Methods

### 2.1. Aggregate Image Acquisition

The ability to rapidly evaluate coarse aggregates in their stacked state on conveyor belts is of great significance for asphalt mixture production, as aggregate shape uniformity directly influences packing density, mechanical interlock, and pavement performance [13]. Traditional laboratory methods typically rely on sieved or manually isolated particles, which fail to capture the variability inherent in bulk aggregates under real production conditions. To address this limitation, the present study employed a laboratory-simulated conveyor belt setup to acquire images of stacked aggregates, ensuring both experimental controllability and preservation of natural particle overlaps for subsequent batch-level analysis.

**Figure 1:**
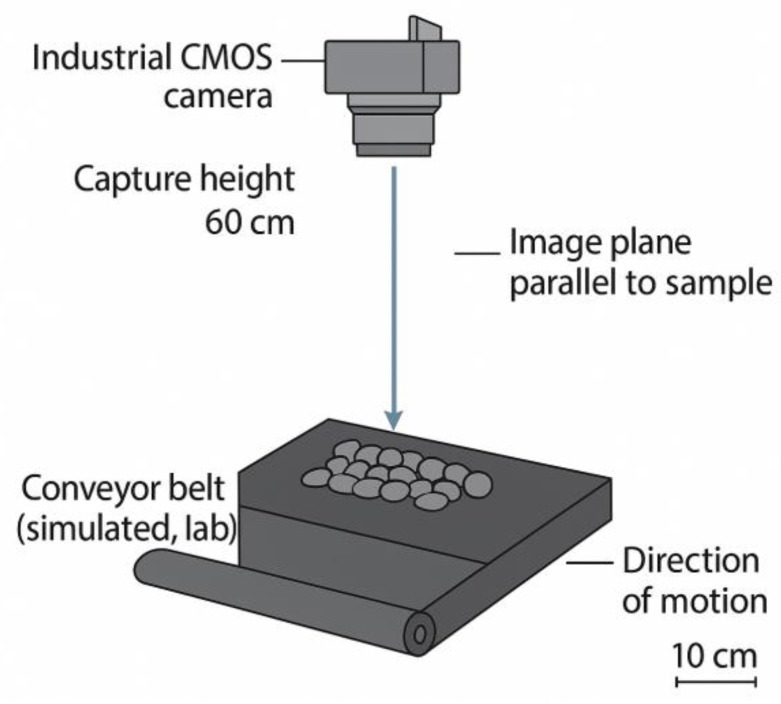
Laboratory-simulated conveyor belt imaging setup for stacked coarse aggregates.

The aggregates investigated were crushed stones commonly used in asphalt pavement construction. Representative samples were randomly collected from stockpiles and gently deposited onto the conveyor belt to maintain a realistic stacked configuration, including random orientations and natural contacts between particles. This design mirrors the bulk distribution encountered in production and enables image-based analysis that is consistent with real operational conditions. The experimental arrangement for on-belt imaging of stacked coarse aggregates is shown schematically in Figure 1.

**Figure 2:**
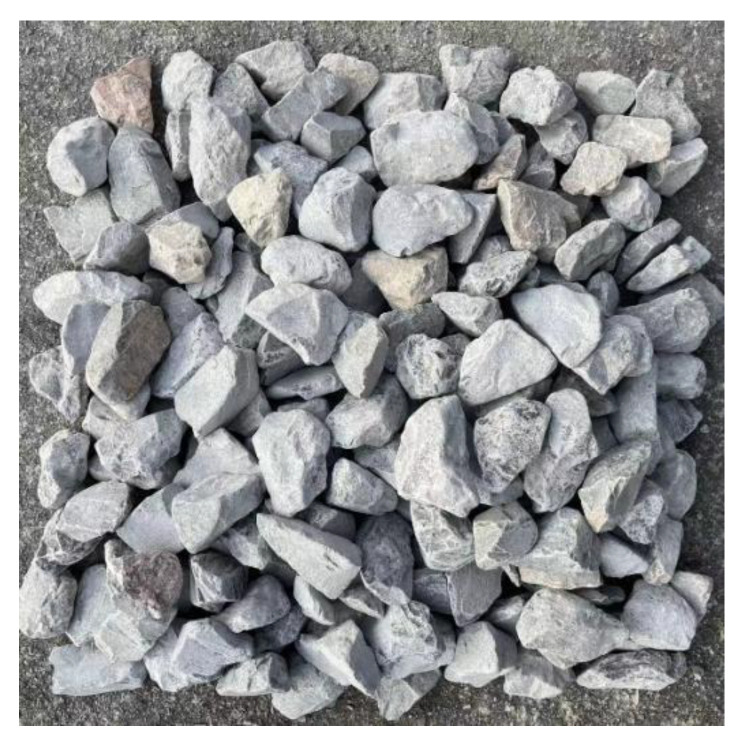
Raw image of stacked coarse aggregates captured in the laboratory.

For image acquisition, a MindVision industrial CMOS area-scan camera (model MV-GE500M-T-CL, MindVision, Shenzhen, China) equipped with a 12 mm fixed-focus lens was mounted 0.60 m above the stacked aggregates. At this working distance, the camera covered a field of view of approximately 230 mm × 230 mm, and grayscale images were recorded at a resolution of 1024 × 1024 pixels, corresponding to a spatial sampling of about 0.225 mm (225 μm) per pixel in the object plane. When illumination was insufficient, auxiliary light sources were employed to ensure boundary clarity and reduce shadow artifacts. These acquisition parameters guarantee high-quality images suitable for subsequent preprocessing, segmentation, and quantitative analysis. A typical raw image of stacked aggregates captured under the laboratory-simulated conveyor belt condition is presented in Figure 2. The image illustrates the natural distribution, random orientation, and frequent particle overlaps that occur in bulk aggregates, highlighting the challenges for boundary detection and the necessity of effective preprocessing and segmentation methods.

The tested materials were crushed coarse aggregates commonly used in asphalt mixtures. For particle-level validation, 30 images of stacked aggregates containing a total of 1050 particles were analyzed, and each particle was measured once manually and once by the proposed image-based method. For batch-level evaluation, 50 additional images of stacked aggregates were acquired under the same imaging conditions, with each image representing one aggregate batch for analysis of statistical shape characteristics.

### 2.2. Image Preprocessing and Segmentation

As shown in Figure 2, the raw stacked-aggregate image exhibits uneven illumination, surface noise, and blurred boundaries caused by particle overlaps. These issues necessitate a series of preprocessing operations to enhance image quality prior to segmentation.

#### 2.2.1. Image Enhancement

To reduce noise and improve boundary clarity in raw aggregate images, a series of enhancement techniques was applied prior to segmentation.

First, the RGB images were converted to grayscale, which simplifies the data structure and enhances the intensity contrast between aggregates and the background. This step suppresses color variations unrelated to particle geometry while emphasizing edge transitions.

Second, histogram equalization was performed to redistribute pixel intensities across the available dynamic range. This operation strengthens the visibility of aggregates located in poorly illuminated regions and highlights subtle edge features that may otherwise be obscured.

Finally, Gaussian filtering was used to suppress high-frequency noise without significantly blurring the main particle contours. This smoothing process reduces spurious edges and prepares the image for stable segmentation.

**Figure 3:**
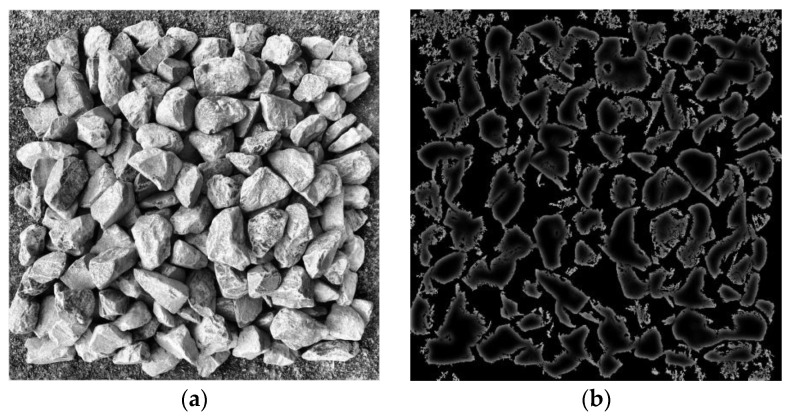
Results of image enhancement: (**a**) histogram equalization; (**b**) Gaussian filtering.

The results of the enhancement process are shown in Figure 3, where (a) illustrates the effect of histogram equalization, and (b) presents the Gaussian-filtered image with reduced noise and clearer contours.

#### 2.2.2. Edge Detection and Segmentation

Following image enhancement, segmentation was conducted to separate individual aggregate particles from the background. The Canny edge detector was first applied to highlight intensity discontinuities corresponding to aggregate boundaries. This method provides high detection accuracy while suppressing noise-induced false edges [14].

However, due to particle overlaps and irregular shapes, simple edge detection was insufficient for complete separation. To address this, the watershed algorithm was employed, which treats the grayscale image as a topographic surface. By simulating the flooding process, watershed segmentation delineates particle boundaries based on local intensity minima [15,16]. The combined approach ensures that adjacent aggregates are separated even when their contours partially overlap.

**Figure 4:**
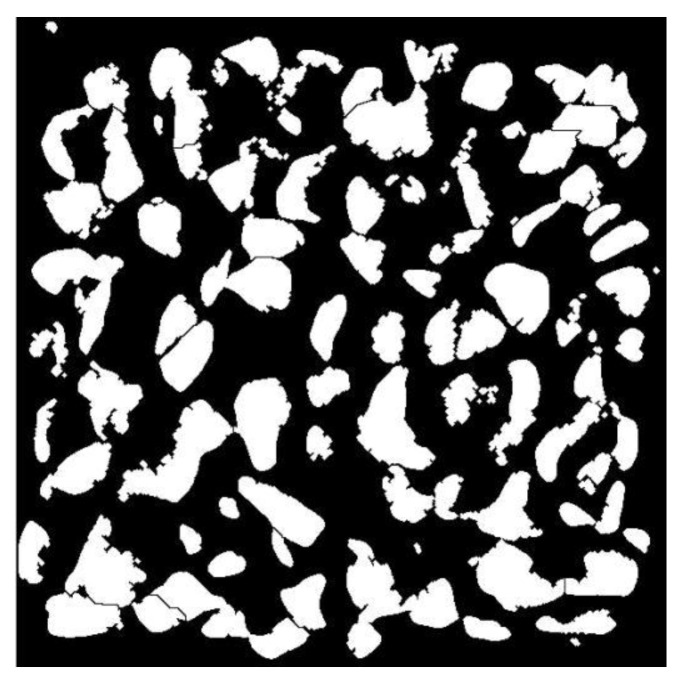
Edge detection and segmentation results of stacked aggregates.

The segmentation results are shown in Figure 4, where individual particles are distinguished with clear boundaries suitable for subsequent shape analysis.

#### 2.2.3. Contour Extraction

**Figure 5:**
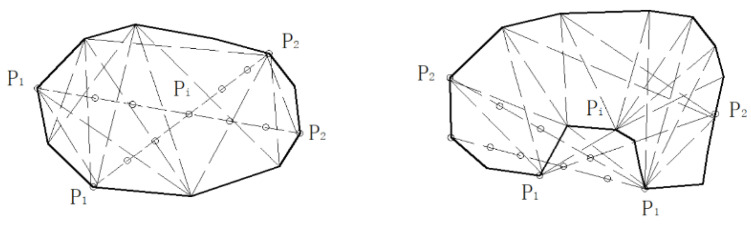
Schematic illustration of the Graham Scan convex hull algorithm, where *P*_1_ and *P*_2_ are boundary points on the particle contour and *P*_i_ is an intermediate boundary point.

To reconstruct particle boundaries after segmentation, the Graham Scan convex hull algorithm was employed [17]. Since most crushed aggregates can be approximated as convex polygons, this approach effectively rebuilds their contours while minimizing segmentation errors. A schematic illustration of the convex hull construction process is shown in Figure 5.

The principle of the convex hull is that for a convex polygon, all vertices lie on the same side of any extended edge line. Based on this property, the Graham Scan algorithm identifies the outermost points of a particle and reconstructs its convex polygonal outline. The steps can be summarized as follows:

(1) Reference point selection: Identify the point P0 with the minimum y-coordinate as the base point.
(2) Polar angle sorting: Connect all other points to P0 and sort them by polar angle relative to the x-axis.
(3) Counterclockwise traversal: Sequentially traverse the sorted points in counterclockwise order, adding points to the convex hull.
(4) Convexity check: Remove intermediate points that violate the convex condition, ensuring the final polygon remains convex.

**Figure 6:**
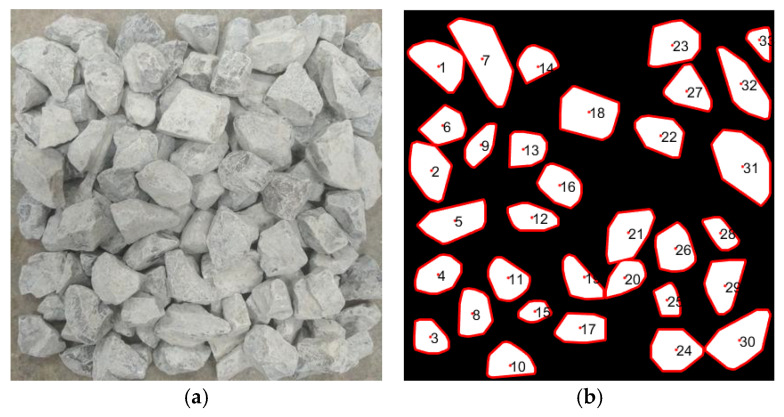
Preprocessing result of aggregate contour reconstruction using the Graham Scan convex hull algorithm. (**a**) Original stacked aggregate image. (**b**) Reconstructed particle contours. The red boundaries denote the convex hulls of individual aggregate particles, and the numbers inside each particle are the identification indices of the particles.

Through iterative updating, the algorithm outputs the maximum convex hull enclosing all particle boundary points. This reconstructed contour ensures that each aggregate is represented as a complete convex polygon, providing reliable input for subsequent morphological evaluation. The preprocessing effect of convex hull reconstruction applied to stacked aggregates is illustrated in Figure 6.

### 2.3. Shape Evaluation Method and Standard

#### 2.3.1. Particle-Level Shape Descriptors

After contour reconstruction, quantitative analysis of individual aggregates was carried out by computing both size- and shape-related descriptors from two-dimensional digital images. The size parameters include the projected area (*S*) and perimeter (*C*), which are the most basic measurements for describing particle geometry.

The area of an aggregate particle (*S*) is defined as the region enclosed by its boundary contour, which corresponds to the total number of pixels within the region in a digital image. The mathematical expression of the area is given in integral form, as shown in Equation (1), where R denotes the region occupied by the aggregate particle.

(1) $$S=\iint_{R}dxdy$$

The perimeter of an aggregate particle (*C*) is defined as the length of its boundary contour, which corresponds to the total number of pixels along the edge in a digital image. The mathematical expression of the perimeter is given in Equation (2).

(2) $$C=\int \sqrt{x^{2}(\theta )+y^{2}(\theta )}d\theta$$

**Figure 7:**
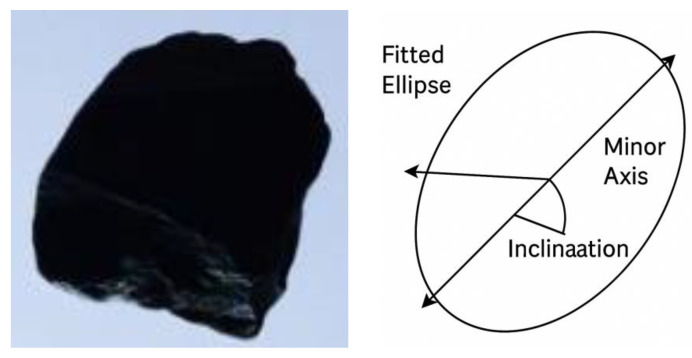
Fitted ellipse of the aggregate particle.

To further capture particle dimensions, the equivalent ellipse method was employed to evaluate the shape dimensions of individual aggregate particles. By applying a least-squares fitting approach, this method provides the maximum length (*L*) and minimum width of the particle (*B*), as well as its perimeter (*C*) and area (*S*). An example of ellipse fitting is given in Figure 7.

On the basis of these geometric primitives, two shape descriptors were derived. The first is the aspect ratio (AR), defined as the ratio of the ellipse length to width (AR = *L*/*B*). A larger AR indicates that the particle tends to be elongated or needle-shaped. The second is the shape factor (F), defined as F = *C*^2^/(4π*S*), where *C* and *S* denote the perimeter and area, respectively. Together, AR and *F* provide an objective quantification of aggregate morphology and establish a robust basis for subsequent batch-level evaluation and quality classification. The shape factor (F) also reflects circularity and characterizes how closely a particle approaches an ideal round shape based on its perimeter and area. Because it integrates multiple geometric parameters and reduces evaluation bias caused by a single measurement feature, F is adopted in this study as a key indicator for describing particle morphology.

#### 2.3.2. Batch-Level Statistical Metrics

To extend particle-level descriptors to the evaluation of aggregate batches, statistical metrics were introduced. For a batch consisting of *n* particles, the mean value of a given shape descriptor ($\bar{F}$) reflects the overall trend of particle morphology, while the standard deviation ($\sigma_{F}$) quantifies the degree of variation within the batch. They are defined as:

(3) $$\bar{F}=\frac{1}{n}\sum_{i=1}^{n}F_{i}$$

(4) $$\sigma_{F}=\sqrt{\frac{1}{n-1}\sum_{i=1}^{n}{(F}_{i}-\bar{F})}$$

Here, F*_i_* represents the descriptor value of the *i*-th particle. The mean provides a global measure of the batch morphology, whereas the standard deviation indicates shape uniformity. A smaller $\sigma_{F}$ implies that the batch exhibits greater consistency in particle geometry, whereas a larger $\sigma_{F}$ highlights significant variability.

#### 2.3.3. Establishment of Evaluation Standard

To provide a practical framework for assessing aggregate shape quality, a classification standard was established based on the statistical metrics defined above. The evaluation standard primarily considers the mean value of key shape descriptors, which reflects the global morphology of the batch. Threshold ranges were determined by analyzing the distribution of shape indices across multiple aggregate samples.

The classification scheme divides aggregate batches into three grades: Grade A (high quality, well-shaped aggregates), Grade B (moderate quality), and Grade C (poor quality, irregularly shaped aggregates). The thresholds were derived from experimental datasets, ensuring that the standard not only reflects numerical differences but also corresponds to visually distinguishable levels of aggregate shape quality.

Furthermore, the use of standard deviation as a supplementary index enhances the robustness of the classification system. For instance, a batch with a favorable mean descriptor value but an excessively high standard deviation would still be penalized, since inconsistent particle morphology can negatively affect compaction and structural performance in pavement applications.

This evaluation standard establishes a clear and reproducible criterion for shape quality assessment, providing the basis for practical implementation in quality control of aggregate production. The detailed application of the standard to experimental datasets is presented in Section 3.

## 3. Results

### 3.1. Particle-Level Results

To verify the reliability of the proposed image analysis method, a comparative study was conducted using 30 groups of stacked aggregate images, involving a total of 1050 individual particles. Each particle was identified by digital image analysis and subsequently measured manually using calipers. The classification results obtained from the two approaches were compared, and the statistical differences are summarized in Table 1.

**Figure 8:**
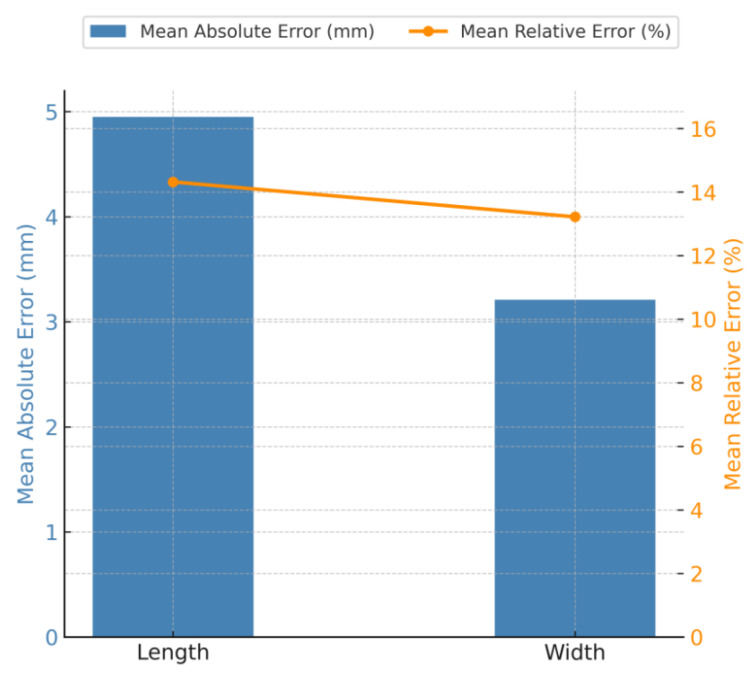
Mean absolute and relative errors of aggregate dimensions.

To better evaluate the reliability of the image-based method, the average absolute error and mean relative error of particle length and width were calculated from the data in Table 1. These results are presented in Figure 8, which provides an intuitive summary of the overall dimensional deviations between manual and image-based measurements.

**Figure 9:**
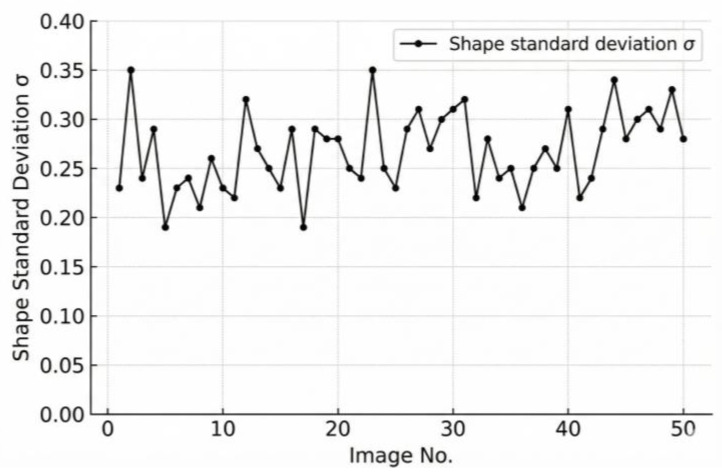
Variation in the shape standard deviation ($\sigma_{F}$) across 50 stacked aggregate images.

As shown in Figure 9, the mean absolute error of particle length was 4.95 mm, while that of particle width was 3.21 mm. The corresponding mean relative errors were 14.32% for length and 13.22% for width. These results indicate that the image-based method achieves a relatively high level of accuracy in capturing both length and width dimensions of stacked aggregate particles. The measurement deviations shown in Table 1 are mainly attributed to three factors: (1) limitations in imaging device accuracy, including intrinsic calibration and sensor resolution; (2) external interference such as illumination variation and imaging noise during acquisition; and (3) the fact that some particles are not perfectly horizontal but tilted when stacked, which results in projection distortion. These reasons lead to differences between image-based measurements and manual readings, although the deviations remain within an acceptable range for engineering applications.

Since this study ultimately adopts the shape factor (F) as the evaluation index instead of absolute geometric dimensions, the influence of such measurement deviations is mitigated. The shape factor normalizes particle geometry using both perimeter and area, making it less sensitive to small dimensional fluctuations. Future improvements may include multi-angle acquisition, automated leveling of particles, or the integration of deep-learning-based contour refinement to further reduce shape distortion and enhance parameter accuracy.

### 3.2. Validation of Batch-Level Statistical Metrics

**Figure 10:**
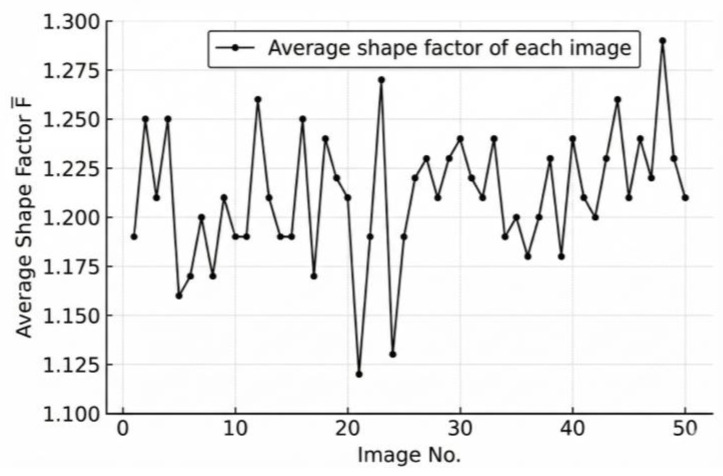
Variation in the average shape factor ($\bar{F}$) across 50 stacked aggregate images.

To provide an overall evaluation of aggregate shape quality at the batch level, 50 images of stacked aggregates were captured. Following the procedure described in Section 2.3.2, both the standard deviation and the mean value of shape factors for the particles in each image were calculated as evaluation indices. These batch-level statistical metrics characterize not only the variability of particle morphology within each image but also the overall tendency of aggregate shape quality across multiple batches. The variation trends of and are illustrated in Figure 9 and Figure 10, respectively.

As shown in Figure 9 and Figure 10, both the shape standard deviation ($\sigma_{F}$) and the average shape factor ($\bar{F}$) exhibit relatively stable trends across the 50 image batches, indicating consistent morphological characteristics of the coarse aggregates. However, the average shape factor $\bar{F}$ primarily reflects the mean geometric feature of all particles within a batch and is therefore insensitive to local variations or abnormal shape distributions. In contrast, the standard deviation $\sigma_{F}$ effectively captures the dispersion of particle shape indices, providing a more representative measure of the overall uniformity of aggregate morphology. Consequently, $\sigma_{F}$ is adopted in this study as the final evaluation indicator for assessing the batch-level shape quality of stacked coarse aggregates.

**Figure 11:**
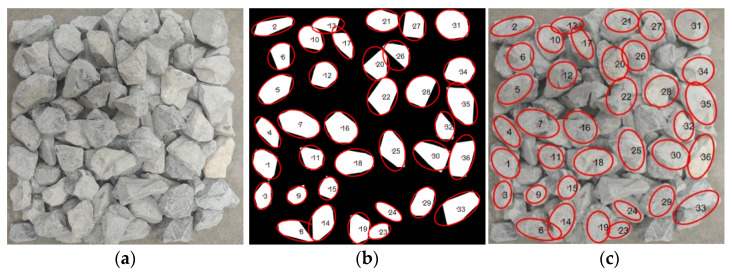
Comparison of sample No. 1 ($\sigma_{F}$ = 0.23). (**a**) original stacked aggregate image; (**b**) segmented image, where red contours indicate the extracted boundaries of individual aggregate particles and the numbers label each particle; (**c**) original image overlaid with the extracted particle boundaries.

**Figure 12:**
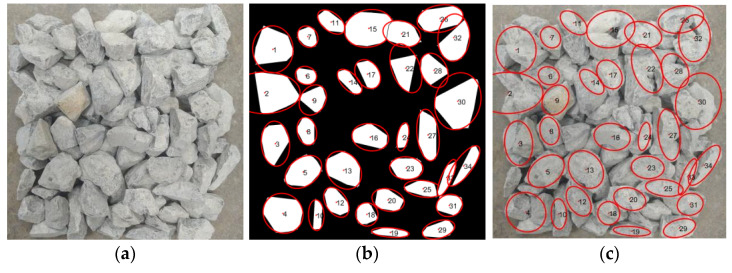
Comparison of sample No. 2 ($\sigma_{F}$ = 0.35). (**a**) original stacked aggregate image; (**b**) segmented image, where red contours indicate the extracted boundaries of individual aggregate particles and the numbers label each particle; (**c**) original image overlaid with the extracted particle boundaries.

**Figure 13:**
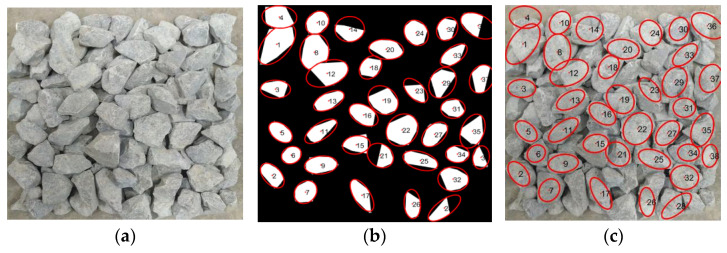
Comparison of sample No. 5 ($\sigma_{F}$ = 0.19). (**a**) original stacked aggregate image; (**b**) segmented image, where red contours indicate the extracted boundaries of individual aggregate particles and the numbers label each particle; (**c**) original image overlaid with the extracted particle boundaries.

As shown in Figure 9 and Figure 10, Image No. 5 exhibits the smallest shape standard deviation ($\sigma_{F}$ = 0.19), indicating the highest shape uniformity, whereas Image No. 2 presents the largest $\sigma_{F}$ value ($\sigma_{F}$ = 0.35), suggesting greater shape variability among particles. Image No. 1 represents a medium condition with $\sigma_{F}$ = 0.23. The corresponding original and processed aggregate images for Image Nos. 1, 2, and 5 are illustrated in Figure 11, Figure 12 and Figure 13, respectively.

As illustrated in Figure 11, Figure 12 and Figure 13, a clear relationship can be observed between the standard deviation of the shape factor ($\sigma_{F}$) and the overall shape quality of the aggregate particles. When $\sigma_{F}$ is small, such as in sample No.5 ($\sigma_{F}$ = 0.19), the particles exhibit more regular geometries with smoother contours and consistent aspect ratios. Conversely, as $\sigma_{F}$ increases, for instance, in sample No. 2 ($\sigma_{F}$ = 0.35), the aggregates display more irregular boundaries, angular edges, and pronounced variability in particle shapes. This trend indicates that the shape factor standard deviation ($\sigma_{F}$) effectively reflects the dispersion of particle morphology within each image, and can thus serve as a quantitative indicator of batch-level shape quality.

Based on the calculated results for all 50 images, the classification criteria for aggregate shape quality can be established as follows:

When $\sigma_{F}$ ∈ [0, 0.32], the aggregate shape quality is excellent;

When $\sigma_{F}$ ∈ (0.32, 0.42], it is good;

When $\sigma_{F}$ > 0.42, the shape quality is considered poor.

This classification provides a clear and objective standard for evaluating the overall morphology of stacked coarse aggregates from digital images.

## 4. Conclusions

This study proposed a digital image-based approach for evaluating the shape characteristics of stacked coarse aggregates and established a quantitative and automated framework for batch-level shape quality assessment.

(1) The developed image-processing pipeline integrates enhancement, Gaussian filtering, contour extraction using the Graham scan convex hull algorithm, and morphological parameter computation, enabling accurate boundary identification even under complex particle overlap conditions.
(2) The morphological characteristics of individual aggregates were quantified using the equivalent ellipse method, in which the shape factor ($\bar{F}$) and its standard deviation ($\sigma_{F}$) were introduced as the key parameters reflecting particle uniformity and overall shape quality. Based on the analysis of 50 stacked aggregate images containing 1050 particles, the results demonstrate that $\sigma_{F}$ effectively characterizes the dispersion degree of particle geometries—smaller $\sigma_{F}$ values correspond to more uniform and better-shaped aggregates, while larger $\sigma_{F}$ values indicate greater shape variability and poorer overall quality.
(3) According to the experimental data, an evaluation criterion for batch-level aggregate shape quality was established. When $\sigma_{F}$ lies between 0 and 0.32, the aggregates exhibit excellent shape uniformity; values between 0.32 and 0.42 correspond to good shape quality; and $\sigma_{F}$ values greater than 0.42 indicate poor shape consistency. This $\sigma_{F}$-based evaluation index provides a reliable and interpretable measure for aggregate shape assessment and can be further extended to real-time monitoring of aggregates on conveyor belts, supporting intelligent quality control in material production and pavement construction.
(4) Although the proposed method demonstrates satisfactory performance in stacked aggregate detection, there is still room for further improvement. The classification thresholds proposed in this study (excellent, good, and poor) were derived based on the observed statistical distribution of the shape factor $\sigma_{F}$ and its consistency with manually measured particle morphology. Although no external benchmark currently exists for stacked aggregate morphology evaluation, the proposed thresholds align with engineering judgment commonly used in asphalt mixture quality control. Moreover, the consistency between the classification results and manual inspection confirms the rationality of the threshold settings. Future work will include external validation using larger datasets and cross-validation with other digital aggregate characterization methods.

## Figures and Tables

**Table 1:** Comparison between image-based and manual measurements of aggregate dimensions.

No.	Actual Length (mm)	Elliptical Major Axis (mm)	Relative Error (%)	Actual Width (mm)	Elliptical Minor Axis(mm)	Relative Error (%)
1	32.15	32.10	0.15	24.65	24.97	1.32
2	37.01	38.91	5.12	22.07	20.47	7.24
3	38.87	28.49	26.70	24.08	20.74	13.87
4	36.76	34.10	7.25	24.72	20.46	17.22
5	39.57	36.41	7.99	29.94	24.60	17.85
6	26.51	29.03	9.50	22.23	25.72	15.68
7	28.15	38.74	37.63	23.96	26.30	9.76
8	33.64	40.22	19.57	22.62	22.31	1.38
9	32.51	24.49	24.67	24.76	20.34	17.83
10	36.09	29.37	18.62	26.68	24.34	8.75
11	30.03	27.73	7.66	24.17	24.08	0.36
12	32.93	29.66	9.93	24.41	26.09	6.89
13	29.68	31.71	6.84	19.8	20.20	2.00
14	37.21	35.13	5.60	23.43	24.80	5.83
15	36.06	25.89	28.21	27.03	21.68	19.81
16	31.95	33.70	5.47	28	28.50	1.79
17	42.15	33.54	20.42	22.05	19.88	9.83
18	39.3	35.86	8.75	26.52	26.89	1.40
19	35.62	30.96	13.07	28.59	24.19	15.38
20	34.67	32.62	5.92	28.35	26.19	7.62
21	39.13	32.27	17.53	30.33	23.74	21.72
22	33.45	35.31	5.56	29.64	28.03	5.43
23	40.33	25.97	35.60	28.11	19.00	32.41
24	35.72	30.25	15.32	27.6	17.84	35.36
25	34.58	37.25	7.73	32.56	25.47	21.76
……	……	……	……	……	……	……
1046	36.79	43.13	17.22	29.94	30.23	0.95
1047	30.02	39.64	32.04	24.09	29.83	23.84
1048	48.99	38.90	20.61	30.42	25.90	14.86
1049	37.45	31.97	14.64	23.57	22.85	3.06
1050	39.4	35.49	9.92	20.11	23.97	19.21

Note: “…” indicates that intermediate rows (No. 26–1045) are omitted.

## Data Availability

The data that support the findings of this study are available from the corresponding author upon reasonable request.

## References

[1] Li X., Shi L., Liao W., Wang Y., Nie W. (2024). Study on the Influence of Coarse Aggregate Morphology on the Meso-Mechanical Properties of Asphalt Mixtures Using Discrete Element Method. Constr. Build. Mater..

[2] Gawenda T., Surowiak A., Krawczykowska A., Stempkowska A., Niedoba T. (2022). Analysis of the Aggregate Production Process with Different Geometric Properties in the Light Fraction Separator. Materials.

[3] Stempkowska A., Gawenda T., Naziemiec Z., Ostrowski K.A., Saramak D., Surowiak A. (2020). Impact of the Geometrical Parameters of Dolomite Coarse Aggregate on the Thermal and Mechanical Properties of Preplaced Aggregate Concrete. Materials.

[4] Roshan A., Abdelrahman M. (2024). Influence of Aggregate Properties on Skid Resistance of Pavement Surface Treatments. Coatings.

[5] Wang L., Yao Y., Li J., Tao Y., Liu K. (2022). Review of Visualization Technique and Its Application of Road Aggregates Based on Morphological Features. Appl. Sci..

[6] Castillo D., Pouteau P., Suquet P., Vanel L. (2024). Image-Based Gradation and Aggregate Characterisation: Case of Cement-Stabilised Quarry Fines. Road Mater. Pavement Des..

[7] Li M., Liu X., Ni X., Koukkari P., He Y. (2022). Analysis of Particle Size Distribution of Coke on Blast Furnace Belt Using Object Detection. Processes.

[8] Dong Y., Wang Z., Ren W., Jiang T., Hou Y., Zhang Y. (2023). Influence of Morphological Characteristics of Coarse Aggregates on Skid Resistance of Asphalt Pavement. Materials.

[9] Xiao Y., Peng Y., Wang M., Ning Y., Zhou Y., Kong K., Long Y. (2024). A Novel Method for Predicting Coarse Aggregate Particle Size Distribution Based on Segment Anything Model and Machine Learning. Constr. Build. Mater..

[10] Christie G., Hyndman A., Moyle S. (2015). Fast Inspection for Size-Based Analysis in Aggregate Production Using 2D Vision. Mach. Vis. Appl..

[11] Sun Z., Li Y., Pei L., Li W., Hao X. (2022). Classification of Coarse Aggregate Particle Size Based on Deep Residual Network. Symmetry.

[12] Vincent L., Soille P. (1991). Watersheds in Digital Spaces: An Efficient Algorithm Based on Immersion Simulations. IEEE Trans. Pattern Anal. Mach. Intell..

[13] Meyer F., Najman L., Talbot H. (2012). The Watershed Concept and Its Use in Segmentation: A Brief History. Mathematical Morphology: From Theory to Applications.

[14] Graham R.L. (1972). An Efficient Algorithm for Determining the Convex Hull of a Finite Planar Set. Inf. Process. Lett..

[15] Igathinathane C., Pordesimo L.O., Columbus E.P., Batchelor W.D., Methuku S.R. (2008). Shape Identification and Particle Size Distribution from Basic Shape Parameters Using ImageJ. Comput. Electron. Agric..

[16] Murtagh F., Basset J.M., Lemaire J. (2005). A Machine Vision Approach to the Grading of Crushed Aggregate (On-belt Camera). Mach. Vis. Appl..

[17] Wang H., Luo D., Zhang K., Chen X. (2023). A deep learning-based framework for detecting coarse aggregate morphology in asphalt mixtures. Autom. Constr..

